# Festina Lente

**Published:** 1884-07

**Authors:** Geo. E. Blackham


### FESTINA LENTE.

GEO. E. BLACKHAM, M. D.

"Make haste slowly," good advice that and much needed, though little heeded in these rushing, pushing days,—when the chief end of existence seems to be to spend it as rapidly as possible. In school the child is crowded forward as rapidly as possible, as many things as possible are studied in as short a time as possible, and the graduate is sent out into the world with a smattering of many things, but no thorough knowledge of anything and, too often, with a broken constitution into the bargain. Less haste, a longer period of study; less study out of school hours, a few subjects well mastered rather than many things hastily skimmed through, would have been a better preparation for the battle of life.

In business it is the same—the old slow way of *earning* a competence won't answer now. Riches must be reached by a shorter road. The broker's office holds out its bait of speculation on margins—a lucky "strike" convinces the clerk or book-keeper that he is on the road to wealth and he re-invests his winnings, then the market goes against him and he "borrows" some of his employer's funds to keep up his margins, and then when the crash comes, as it is sure to, he is not only a beggar but a criminal. It would have been better for him to have "made haste slowly."

The banker with a fine business is not content, though the dollars roll into his coffers by the hundreds. Legitimate business is "too slow" and so he goes into Wall street as a "bull" or a "bear," the money of the merchant, the widow and the orphan has been intrusted to him and he wagers it on the issue of a blind pool and when he loses, he ruins not only himself and his partners, but hundreds of innocent depositors who had trusted in his honesty and business integrity He exchanges a palace on Fifth avenue for a cell in jail and wishes, when too late, that he had made haste more slowly.

The young man whose glory is his strength is not content to be strong and swift, he must be stronger and swifter than someone else. He rows in the university regatta, he "beats the record" and falls fainting into the arms of his comrades as he crosses the finish line. The delicate mechanism which controls the circulation of his blood has been strained beyond endurance and never again can he take part in any athletic exercise. Had he made haste slowly his exercise would have increased his strength, and aided to give him a hale and hearty old age.

Mr. H. L. Cortis, a young English gentleman, and a student of medicine, developed great speed on the bicycle, and took part in numerous amateur races with such success that when a year ago he took his degree as member of the Royal College of Surgeons, retired from the path, married and went to Australia to begin his professional life, he held the amateur records from one to twenty-five miles. and on two occasions at least had covered twenty miles within the hour. He did not make haste slowly and retired in a blaze of glory, and now as might have been expected, the cycling papers are publishing notices to the following effect :

"We regret to learn that H. L. Cortis, Esq., M. R. C. S., late amateur champion bicycler, is lying dangerously ill of heart disease at Sydney, Australia."

Of how much consolation to him or his young wife in a strange land, will it be that he made haste more rapidly than any other man in the world if the result is to be his taking off in the morning of his professional life and usefulness.

The mechanic would be a journeyman without the delay of apprenticeship, the student a doctor with a year or two of study, and when the boiler bursts because of bad workmanship, or the patient dies because of ignorant treatment, we call it a dispensation of providence instead of recognizing it as the inevitable result of failing to "make haste slowly."

This is the age of haste. The express trains that are most to be desired are those which make the fewest stops. The telegraph and the telephone anihilate time and space.

Childhood is out of fashion, the babe is a man or a woman at an age when its slower parents were boy and girl.

The great strife is not to do something well but to do many things rapidly, to do more things in a given time or some one thing faster than someone else, to rush, rush, rush, hurry, hurry, hurry, till all the comfort, all the poetry, all the excellence is taken out of life, and humanity seems like the Mississippi river steamer with a hogshead of bacon in the firebox and a "nigger" on the safety valve, rushing headlong to destruction unmindful of the sunken snag in the river and the impending explosion of the boiler, if only the other boat may be beaten in the race. How many brains have broken down to softening, how many bright intellects have gone into eclipse, how many bodies have been destroyed and hearts broken in this insane race after a bubble. Sloth is a bad thing, but over haste is equally bad. Now as of old, it is only "in the middle way there is safety," and therefore I say unto you, "Make haste slowly."

